# Heteroplasmy and Copy Number in the Common m.3243A>G Mutation—A Post-Mortem Genotype–Phenotype Analysis

**DOI:** 10.3390/genes11020212

**Published:** 2020-02-18

**Authors:** Leila Motlagh Scholle, Stephan Zierz, Christian Mawrin, Claudia Wickenhauser, Diana Lehmann Urban

**Affiliations:** 1 Department of Neurology, Martin-Luther-University Halle-Wittenberg, 06120 Halle (Saale), Germany; stephan.zierz@medizin.uni-halle.de; 2 Institute of Neuropathology, Otto-von-Guericke University, 39120 Magdeburg, Germany; christian.mawrin@med.ovgu.de; 3 Institute of Pathology, Martin-Luther-University Halle-Wittenberg, 06108 Halle (Saale), Germany; claudia.wickenhauser@uk-halle.de; 4 Department of Neurology, University of Ulm, 89081 Ulm, Germany; diana.lehmann@rku.de

**Keywords:** MELAS-syndrome, genotype–phenotype correlation, mtDNA heteroplasmy, m.3243A>G

## Abstract

Different mitochondrial DNA (mtDNA) mutations have been identified to cause mitochondrial encephalopathy, lactate acidosis and stroke-like episodes (MELAS). The underlying genetic cause leading to an enormous clinical heterogeneity associated with m.3243A>G-related mitochondrial diseases is still poorly understood. Genotype–phenotype correlation (heteroplasmy levels and clinical symptoms) was analysed in 16 patients (15 literature cases and one unreported case) harbouring the m.3243A>G mutation. mtDNA copy numbers were correlated to heteroplasmy levels in 30 different post-mortem tissue samples, including 14 brain samples of a 46-year-old female. In the central nervous system, higher levels of heteroplasmy correlated significantly with lower mtDNA copy numbers. Skeletal muscle levels of heteroplasmy correlated significantly with kidney and liver. There was no significant difference of heteroplasmy levels between clinically affected and unaffected patients. In the patient presented, we found >75% heteroplasmy levels in all central nervous system samples, without harbouring a MELAS phenotype. This underlines previous suggestions, that really high levels in tissues do not automatically lead to a specific phenotype. Missing significant differences of heteroplasmy levels between clinically affected and unaffected patients underline recent suggestions that there are additional factors such as mtDNA copy number and nuclear factors that may also influence disease severity.

## 1. Introduction

Mitochondria are involved in various cellular functions, including energy production, metabolic regulation, apoptosis, calcium homeostasis, cell proliferation and motility, as well as free radical generation [1]. Mostly, mitochondria are called the “powerhouse of the cell” as they provide up to 90% of a cell’s adenosine triphosphate (ATP) [2]. The human mitochondrial DNA (mtDNA) is a double-stranded 16.5 kb circle molecule. The mtDNA encodes for 13 essential subunits of the mitochondrial respiratory chain unit, 2 ribosomal mt-RNAs (rRNAs) and 22 mt-tRNAs, which are used for translation of those 13 polypeptides [2,3]. Therefore, mtDNA-mutations may cause respiratory chain (OXPHOS) deficiencies. This might affect any organ, but tissues with high energy consumption, such as brain and muscle, suffer most [4]. Mitochondrial diseases are associated with a wide spectrum of clinical phenotypes, ranging from mild to severe [5], and can either be caused by mutations in the mitochondrial DNA (mtDNA) itself or due to mutations of nuclear origin: (I) nuclear genes encoding for enzymes involved in mitochondrial nucleotide synthesis (*TK2, SUCLA2, SUCLG1, RRM2B, DGUOK* and *TYMP*) or (II) those required for mtDNA replication (*POLG* and *TWNK*) [6]. 

In patients with mtDNA mutations (e.g., m.3243A>G), mutant and wild-type mtDNA molecules coexist within the same cell, which is called heteroplasmy [4]. Patients with higher heteroplasmy levels tend to have more severe disease burden and progression rate; however, disease burden and progression vary greatly between individuals, and the associated clinical spectrum is broad [7]. Furthermore, tissue segregation patterns of m.3243A>G vary widely. Post-mitotic tissues such as muscle have higher levels of mutant mtDNA, whilst levels in blood decrease significantly over time [8]. The mtDNA is present at hundreds to thousands of copies per cell in a tissue-specific manner [1]. mtDNA copy number also varies during aging and disease progression and therefore might be considered as a biomarker that mirrors alterations within the human body [1]. 

MELAS (mitochondrial encephalopathy, lactate acidosis and stroke like episodes) Online Mendelian Inheritance in Man (OMIM) #540000 is one of the most common neurological diseases of child or adult onset [9]. Approximately 80% of patients with MELAS carry the m.3243A>G point mutation in the *MT-TL1* gene (encoding mt-tRNA^Leu(UUR)^ [10,11]. The complexity (heteroplasmy and mt-DNA copy number) of mitochondrial diseases explains why no clear genotype–phenotype correlation has been established yet.

Recently, it has been suggested that even nuclear genetic factors might influence the clinical outcome in m.3234A>G-related diseases [8]. A negative significant correlation of skeletal muscle mtDNA copy number with reduced total disease burden has been reported [7]. The same study demonstrated that, although a substantial amount of variation in m.3243A>G-related disease burden remains to be explained, the best correlates are skeletal muscle mtDNA copy number, heteroplasmy and age [7]. 

The determination of heteroplasmy and mtDNA copy number in all affected tissues of patients would be useful to further investigate a possible correlation of these factors and disease severity. However, the availability of post-mortem tissue is limited. The aim of this study was to investigate a possible correlation between mtDNA copy number and heteroplasmy levels in different tissues with regard to the clinical phenotype. In a post-mortem analysis, we determined the mtDNA/nuclear DNA (nDNA)- ratio and the level of heteroplasmy in 30 different tissue samples of a 46-year-old female (non-MELAS phenotype), including 14 samples of the central nervous system. Furthermore, we collected data from 15 literature cases for further genotype–phenotype analysis.

## 2. Materials and Methods

### 2.1. Patient

A 46-year old Caucasian woman presented with a recently diagnosed m.3243A>G mutation (60% level of heteroplasmy in the biceps brachii muscle) without harbouring a MELAS phenotype. Clinical findings included optic neuropathy, sensorineural hearing loss and diabetes mellitus type I. Stroke-like episodes were not reported. Her medical history included intermittent atrial fibrillation, ketoacidosis, pneumogenic sepsis, liver and kidney failure, cor pulmonale and tricuspid and mitral regurgitation. Family history revealed no evidence for neuromuscular disorders. Clinical examination showed pronounced sensorineural hearing loss, generalized tetra paresis (MRC 2–3/5), and muscle tendon reflexes could not be obtained. Serum creatine kinase (CK) levels were normal. Neurophysiological examinations showed evidence for critical-illness polyneuropathy without signs for acute Guillain–Baré Syndrome. Lumbal puncture and brain magnetic resonance imaging (MRI) were not performed, due to the poor general condition. 

After 30 days, the patient developed distinct hypokalaemia. In case of suspected sepsis with hypotension and increasing inflammatory values, the patient was transferred to the neurological intensive care unit. The patient developed a rapid state of shock with pronounced lactate acidosis, acute kidney and liver failure and hypothermia (33°). Thorax and abdominal CT (computed tomography) scan showed no sepsis focus. Calculated antibiosis was initiated, among the inflammatory values, and improved significantly. On haemodialysis, lactate acidosis was also declining. However, the cardiopulmonary situation worsened, and the patient became catecholamine dependent. In addition, the liver enzymes increased, and a plasmatic coagulation was no longer detectable. Echocardiography showed cardiomegaly with decreasing ejection fraction. In the further course, the patient was in need of reanimation, but could be stabilized. The patient quickly developed a pulseless electrical activity, which caused death after resuscitation. Autopsy revealed an acute right-left heart failure with multi-organ failure after re-edivating pulmonary embolisms, starting from a multi-stage phlebothrombosis and a parietal thrombosis of the right atrium. Aggravating was an advanced high-grade coronary atherosclerosis with more than 90% stenosis in all main vessels and evidence of diffuse myocardial scars and multi-organ failure, as well as florid purulent bronchopneumonia.

### 2.2. Literature Cases

Based on a Pubmed literature search, 15 patients harbouring the m.3243A>G were included in this study according to their clinical description and post-mortem heteroplasmy levels (Table 1). All patients included were autopsied and several levels of heteroplasmy (m.3243A>G) of different tissues were analysed post-mortem (PM).

Details of PM mtDNA heteroplasmy analysis from the literature cases (L1–L15) are given in Table 1. If heteroplasmy levels from the quadriceps muscle (used as PM skeletal) were not given, heteroplasmy levels from the psoas muscle were used for further analysis. However, Hamazaki et al. (L12) [12] named the rectus abdominis muscle as skeletal muscle. Brain heteroplasmy levels were taken either from samples declared as the visual cortex or occipital cortex. Heart heteroplasmy levels were either taken from the left ventricle or, in three cases (L13–L15), from the right ventricle.

### 2.3. Muscle Histopathology-Patient

Cryostat sections were cut from transversely orientated muscle blocks from the quadriceps muscle and subjected to standard histological and histochemical analysis including cytochrome c oxidase (COX), succinate dehydrogenase (SDH) and COX–SDH oxidative enzyme reactions.

### 2.4. Restriction Fragment Length Polymorphism (RFLP)-Polymerase Chain Reaction (PCR)-Patient

Total DNA was extracted from different samples using the peqGOLD tissue DNA Mini Kit (Peqlab Biotechnologie GmbH, Erlangen, Germany) according to the manufacturer’s instructions. For RFLP analysis, a 330 bp PCR product spanning the mtDNA 3243 locus was amplified using a forward primer 5′ GGT TCG TTT GTT CAA CGA TT 3′ and a reverse 5′ TGC CAT TGC GAT TAG AAT GG 3′. The PCR product was digested by ApaI (New England Biolabs GmbH (NEB) Frankfurt am Main, Germany) into two fragments of 117 and 213 bp. The digested PCR products were separated on agarose gel and stained with ethidium bromide. For analysis of signal intensity of the bands, ImageJ software was used. 

### 2.5. Determination of mtDNA Copy Numbers-Patient

Total DNA was extracted from different tissues using the peqGOLD tissue DNA Mini Kit (Peqlab Biotechnologie GmbH, Erlangen, Germany), following the manufacturer’s instructions. The concentration and purity of the extracted DNA were determined using a plate reader (Tecan Infinite M200^®^ PRO, Tecan Deutschland GmbH, Crailsheim, Germany). The DNA samples were diluted to 5 ng/μL. 

Relative levels of mtDNA copy number were determined by real-time PCR using singleplex Taqman assays targeting the mitochondrial *MT-ND1* gene (*Hs02596873*) and to a single housekeeping nuclear gene (18 S ribosomal-5-RNA (18S5, *Hs03928990*)). On each 96-well plate, a standard dilution series for every gene to determine absolute copy numbers was amplified in parallel. For each 10-μL single-well reaction contained 5 ng of extracted DNA and 5 μL of TaqMan Universal PCR Master Mix II, No UNG (uracil-N-glycosylase), 0.5 μL TaqMan Assay on the StepOne System (Applied Biosystems, Darmstadt, Germany). The following thermal profile was used: 95 °C for 10 min, 40 cycles of 95 °C for 15 s, 55 °C for 15 s, and 60 °C for 1 min. Each reaction was performed in triplicate. The mtDNA/nDNA ratio was calculated using the 2 (^ΔC^T). 

### 2.6. Ethical Statement

The legal guardian of the patient gave written informed consent for autopsy and inclusion before participation in the study. The study was conducted in accordance with the Declaration of Helsinki and was approved by the Ethics Committee of the University Halle-Wittenberg (Project identification code 215/20.01.10/3).

### 2.7. Statistical Analysis

Statistical analysis, calculation and visualisation were performed using Prism 7 (GraphPad, San Diego, CA, USA). The analysis of a possible relationship between two variables was carried out using linear regression and the Pearson correlation. Analysis of correlation was carried out using multiple *t*-tests. The Holm-Sidak method was used to correct for multiple comparisons. The *p*-value selection was based on the FDR (False Discovery Rate). The value of α was determined at 0.05. Significance was set to *p* = 0.05. The statistical tests chosen were predetermined by the size of the study group and numeric range of values. 

## 3. Results

### 3.1. Muscle Histopathology (Patient)

Muscle biopsy showed myopathic changes, with 5% COX negative fibres and scattered ragged-red-fibres (RRF).

### 3.2. Analysis of Heteroplasmy Levels and Copy Number (Patient)

**Figure 1:**
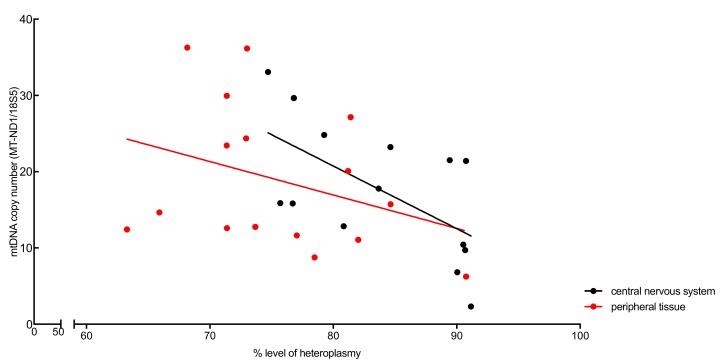
Correlation of heteroplasmy levels (%) and mtDNA copy number (MT-ND1/18S5) in the central nervous system tissue (black circles) and in the peripheral tissues (red circles) from the case report patient. In both the central nervous system samples and the peripheral tissues, higher levels of heteroplasmy correlated with lower mtDNA copy number, however only significantly in the central nervous system samples (*p* < 0.05).

To evaluate a possible correlation between the mtDNA copy number and the heteroplasmic grade in affected organs (14 central nervous system samples and 16 samples from peripheral tissue), the mtDNA/nDNA ratio was calculated as described previously. Heteroplasmy levels (%) and mtDNA copy number (MT-ND1/18S5) were correlated (Figure 1). In both the central nervous system samples and the peripheral tissue samples, higher levels of heteroplasmy correlated with lower mtDNA copy numbers, however only significantly (*p* < 0.05) in the central nervous system samples. 

### 3.3. Analysis of Clinical Phenotype and Genotype in the Patient and Literature Cases

**Figure 2:**
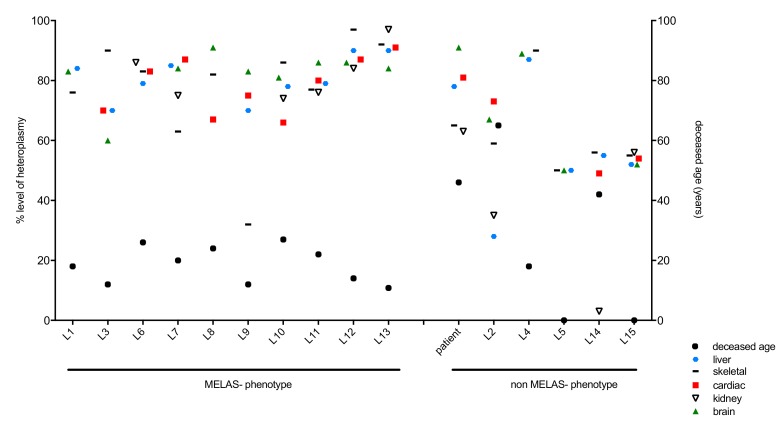
m.3243A>G heteroplasmy levels across literature cases with regard to tissues and clinical phenotype MELAS (mitochondrial encephalopathy, lactate acidosis and stroke-like episodes) versus non-MELAS.

Out of the literature cases, 10 patients were diagnosed with MELAS phenotype (L1, L3, L6–L13), and five patients did not match the MELAS phenotype (L2, L4, L5, L14, L15) (Figure 2, Table 1). In the MELAS cohort, three patients were male, six were female and in one case (L7), the gender was not stated. Deceased age ranged from 10.8 to 27 years (median: 18.58 years). In the non-MELAS cohort, four female patients were included and again, in one case (L15), the gender was not stated. Deceased age ranged from 0 to 65 years (median: 28.53 years). Two patients were stillbirths (L5 and L15).

**Figure 3:**
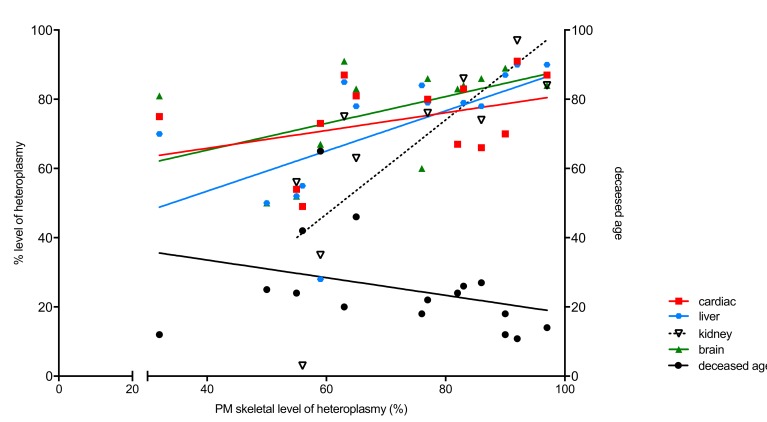
Correlation of heteroplasmy levels (%) in skeletal muscle to levels in cardiac (*p* = n.s.), liver (*p* = 0.0156), kidney (*p* = 0.0100) and brain (*p* = n.s.), plotted on the left y-axis. Correlation of heteroplasmy levels (%) in muscle to deceased age (*p* = n.s.) is plotted on the right y-axis. n.s.: no significant.

mtDNA heteroplasmy (%) levels of all literature patients (L1–L15) and the patient (Table 2) presented in this study were analysed. Skeletal muscle heteroplasmy was correlated to either cardiac, liver, kidney or brain heteroplasmy (Figure 3). In skeletal muscle, heteroplasmy correlated significantly with kidney (*p* = 0.0100) and liver (*p* = 0.0156). There was no significant (n.s.) correlation of cardiac and brain to skeletal heteroplasmy. Skeletal muscle heteroplasmy correlated negatively with deceased age, however not significantly (*p* = 0.2203). Higher deceased age correlated with lower heteroplasmy, however only significantly in liver (*p* < 0.005) and kidney (*p* < 0.05). 

**Figure 4:**
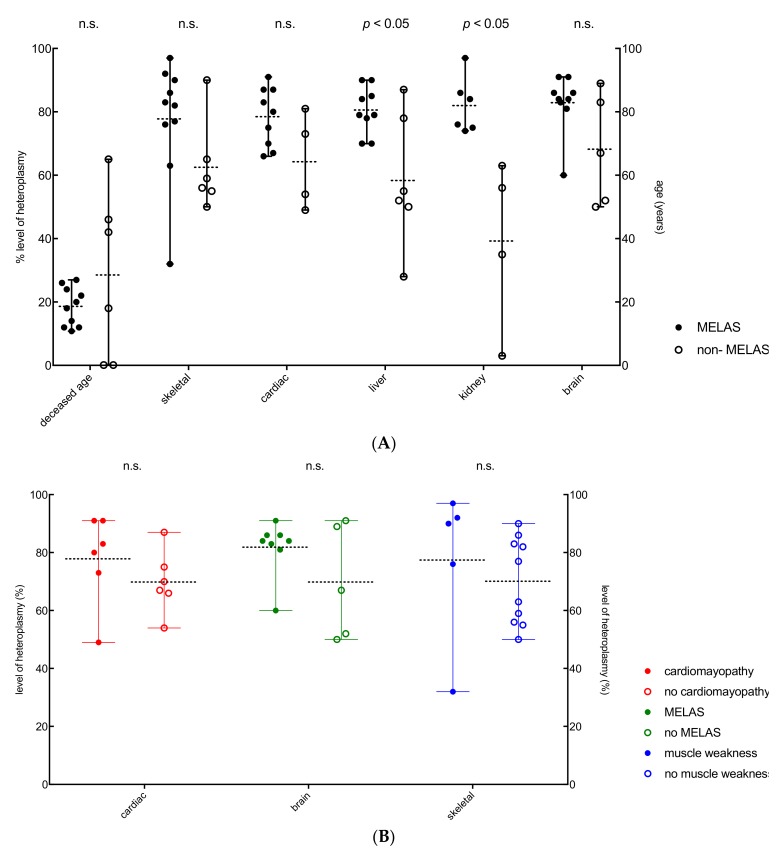
(**A**) Heteroplasmy levels (%) of skeletal, cardiac and brain between MELAS and non-MELAS phenotype patients. Liver and kidney heteroplasmy levels were significantly different in MELAS (closed circles) and non-MELAS (open circles) phenotype patients. Liver (*p* < 0.05) and kidney (*p* < 0.05) heteroplasmy levels were significantly higher in the MELAS patients. (**B**) Heteroplasmy levels (%) of cardiac, brain and skeletal in clinically affected (closed circles) and unaffected patients (open circles), showing no significant difference between affected and unaffected patients.

There was no significant difference of skeletal, cardiac and brain heteroplasmy in deceased age between MELAS (L1, L3, L6–L13) and non-MELAS phenotype (patient, L2, L4, L5, L14, L15) patients (Figure 4A and Table 1). Liver and kidney heteroplasmy levels were significantly different in MELAS and non-MELAS phenotype patients. In both liver (*p* < 0.05) and kidney (*p* < 0.05), heteroplasmy levels were significantly higher in the MELAS cohort (Figure 4A, Table 3). Correlation of heteroplasmy levels (%) of cardiac, brain and skeletal in clinically affected and unaffected patients (Figure 4B) showed no significant difference of heteroplasmy levels in clinically affected and unaffected patients.

## 4. Discussion

Studies of mitochondrial diseases revealed a wide variability in the phenotypic presentation of mitochondrial genetic defects [24]. However, the underlying genetic cause leading to an enormous clinical heterogeneity associated with m.3243A>G-related mitochondrial diseases is still poorly understood [8]. The clinical manifestation of the genetic defect occurs when a threshold level is exceeded [24,25]. Due to heteroplasmy, mtDNA mutant levels can vary dramatically between different individuals and tissues [8]. It is already known, that postmitotic tissues, e.g., muscle, intend to have higher heteroplasmy levels, whilst levels in blood decrease significantly over time [9,11,26,27]. 

Quantification of the abundance of mtDNA molecules harbouring wild-type and pathogenic mutations is important to understand disease progression of mitochondrial disorders and for evaluating therapeutic approaches [3]. Reduction of muscle mtDNA copy number in patients with more severe disease might be due to lower activity levels, which may reduce the mtDNA copy number. Another explanation for this might be a compensatory mechanism of mitochondrial biogenesis in cells harbouring the mutant mtDNA [7]. 

The mtDNA/nDNA ratio of the presented patient was calculated to correlate the mtDNA copy number with the heteroplasmic grade in affected organs. It has been reported that patients with identical heteroplasmy levels can exhibit very different symptoms, and some patients with high heteroplasmy levels are relatively asymptomatic [8]. In the patient presented in this study, higher levels of heteroplasmy correlated with lower mtDNA copy numbers, however only significantly in central nervous system samples (*p* < 0.05). The absolute levels of wild-type (WT) mtDNA have been mentioned as an important indicator for pathological manifestations. [28]. Based on this assumption, selective increase of mtDNA copy number by gene or pharmacological therapy have been suggested as potential treatments for human mtDNA mutation diseases. Interestingly, the patient presented had >75% heteroplasmy levels in all central nervous system samples analysed, without harbouring a MELAS phenotype. This underlines previous suggestions, that really high levels in tissues do not automatically lead to a specific phenotype. 

Recently, it has been evaluated as to which commonly assayed tissue (blood, urine, skeletal muscle) represents the m.3243A>G mutation load and mtDNA copy number most strongly associated with disease burden and progression [7]. Out of this data, it has been suggested that age-corrected blood heteroplasmy is the most convenient and reliable measure for routine clinical assessment in m.3243A>G patients. Interestingly, we found that liver and kidney heteroplasmy correlated significantly with heteroplasmy levels in muscle, however the presented results arose from post-mortem tissue analysis. Furthermore, we did not find a significant difference of heteroplasmy levels in clinically affected and unaffected patients. This underlines recent findings, suggesting that there are additional factors such as mtDNA copy number and nuclear factors that may also influence disease severity [7,8]. The effect of continuous changes of mtDNA heteroplasmy on remodelling of nuclear DNA and mtDNA gene expression profiles has been reported. This effect might be a result of alterations in signal transduction as well as epigenetic regulatory processes. Hence, in contrast to the rules of Mendelian genetics, the heteroplasmy level of a single mtDNA gene could lead to a multiplicity of cellular phenotypes [29,30].

In conclusion, our data highlights previous suggestions, that really high levels in tissues do not automatically lead to a specific phenotype. Missing significant differences of heteroplasmy levels in clinically affected and unaffected patients, underline the assumption that there are additional factors such as mtDNA copy number and nuclear factors that may influence disease severity in an underestimated way.

## 5. Limitations

We are aware that the present study has its limitations. The different sample size (MELAS versus non-MELAS cohort) may influence the statistical impact, especially the wide range of deceased age in the non-MELAS cohort. It must also be noted that the levels of heteroplasmy were analysed in different laboratories. An equal standard of the techniques used cannot be granted. However, the access to post-mortem tissue in a rare mitochondrial disease is limited. Moreover, it needs to be stated that mtDNA copy number was not measured in the literature cases. Due to this, comparison and correlation of heteroplasmy in different tissues is limited and may as well be caused by different cell types or mtDNA copy number. 

## Figures and Tables

**Table 1:** Details of the literature cases included in this study, including sex, clinical information and methods of mitochondrial DNA (mtDNA) heteroplasmy determination; n.k. = not known.

Literature Case Number	Reference	Sex	MELAS-Phenotype	Deceased Age (Years)	Methods of mtDNA Heteroplasmy Determination
*L1*	Prasad et al. 2014 [13]	Male	Yes	18	PCR, restriction enzyme ApaI; quantitative densitometry
*L2*	Prasad et al. 2014 [13]	Female	No	65	PCR, restriction enzyme ApaI; quantitative densitometry
*L3*	Enter et al. 1991 [14]	Female	Yes	12	PCR, restriction endonuclease ApaI; mutation detection by PCR
*L4*	Fornuskova et al. 2008 [15]	Female	No	18	PCR, restriction enzyme ApaI; quantified with ImageQuant software
*L5*	Cardaioli et al. 1999 [16]	stillbirth	No	Miscarriage (25 weeks)	PCR, restriction enzyme ApaI, autoradiography–quantified with ultra-scan densitometer
*L6*	Ciafaloni et al. 1991 [17]	Male	Yes	26	PCR, restriction enzyme HaeIII, quantified with Betascope 603 blot analyser.
*L7*	Kobayashi et al. 1992 [10]	n.k.	Yes	20	PCR, restriction enzyme ApaI; autoradiography, quantified with Fujix bioimaging analyser (BAS2000)
*L8*	Macmillan et al. 1993 [18]	Female	Yes	24	PCR, restriction enzyme ApaI; quantified on a molecular dyanmics series 4000 phosphorImager
*L9*	Obermaier-Kusser et al. 1991 [19]	Female	Yes	12	PCR, restriction enzyme ApaI; densitometry using Elscript 400 UVR scanner
*L10*	Shiraiwa et al. 1993 [20]	Female	Yes	27	PCR, restriction enzyme ApaI; autoradiography, quantified with Fujix bioimaging analyser (BAS2000)
*L11*	Shoji et al. 1993 [21]	Female	Yes	22	PCR, restriction enzyme ApaI; autoradiography, quantified with a densitometer
*L12*	Hamazaki et al. 1993 [12]	Female	Yes	14	PCR, restriction enzyme ApaI; quantified using laser densitometer
*L13*	Koga et al. 2000 [22]	Male	Yes	10.8	PCR, restriction enzyme HaeIII, quantified using a Betascope 603 blot analyser.
*L14*	Matthews et al. 1994 [23]	Female	No	42	PCR, restriction enzyme ApaI; analysed densitometrically from film negatives
*L15*	Matthews et al. 1994 [23]	n.k.	No	stillborn (24 weeks)	PCR, restriction enzyme ApaI; analysed densitometrically from film negatives

**Table 2:** Post-mortem (PM) mtDNA heteroplasmy (%) levels and mtDNA copy number of the patient presented in this study in tissue from the central nervous system (*n* = 14) and peripheral organs (*n* = 16).

Tissue Type (Origin)	Heteroplasmy Levels (%) by RFLP	mtDNA Copy Number MT-ND1/18S5
***Central nervous system***		
*Putamen*	80.84	12.85
*Caudate nucleus*	79.26	24.81
*Mammillary body*	76.80	29.66
*Hypothalamus*	75.70	15.88
*Thalamus*	74.71	33.06
*Internal capsule*	76.71	15.83
*Globus pallidus*	84.63	23.22
*Visual cortex*	83.67	17.79
*Pons*	90.04	6.81
*Medulla oblongata*	90.75	21.41
*White matter*	90.69	9.72
*Cerebellar vermis*	89.43	21.51
*Spinal cord*	90.53	10.42
*Dorsal root ganglion*	91.16	2.31
***Peripheral organs***		
*Liver*	78.48	8.75
*Bladder*	82.02	11.06
*M. obliqus superior*	72.93	24.35
*M. rectus medialis*	71.37	29.95
*M. rectus superior*	73.02	36.17
*M. rectus inferior*	71.36	23.43
*M. iliopsoas*	68.16	36.29
*M. vastus lat.*	65.91	14.65
*M. pectoralis*	73.68	12.76
*M. biceps brachii*	81.20	20.08
*Tongue muscle base*	84.64	15.73
*Heart muscle (left)*	81.40	27.16
*Heart muscle (right)*	77.04	11.64
*AV-node*	71.38	12.60
*Kidney*	63.28	12.42
*Sural nerve*	90.76	6.25

RFLP: Restriction fragment length polymorphism; AV- node: Atrioventricular node.

**Table 3:** Statistical analysis of heteroplasmy levels (%) of PM skeletal, PM cardiac and PM brain, and deceased age of MELAS and non-MELAS phenotype patients.

	Significant	*p*-Value	MELAS (Mean)	Non-MELAS (Mean)
Deceased Age	No	0.266411820	18.58	28.53
Skeletal	No	0.108486887	77.8	62.5
Cardiac	No	0.059542563	78.44	64.25
Liver	Yes	0.011731624	80.56	58.33
Kidney	Yes	0.006090533	82	39.25
Brain	No	0.059855928	82.89	68.2
